# Complete Genome Sequences of Four Avian Paramyxoviruses of Serotype 10 Isolated from Rockhopper Penguins on the Falkland Islands

**DOI:** 10.1128/genomeA.00472-17

**Published:** 2017-06-01

**Authors:** Iryna V. Goraichuk, Kiril M. Dimitrov, Poonam Sharma, Patti J. Miller, David E. Swayne, David L. Suarez, Claudio L. Afonso

**Affiliations:** a Exotic and Emerging Avian Viral Disease Research Unit, Southeast Poultry Research Laboratory, U.S. National Poultry Research Center, ARS, USDA, Athens, Georgia, USA; b National Scientific Center Institute of Experimental and Clinical Veterinary Medicine, Kharkiv, Ukraine

## Abstract

The first complete genome sequences of four avian paramyxovirus serotype 10 (APMV-10) isolates are described here. The viruses were isolated from rockhopper penguins on the Falkland Islands, sampled in 2007. All four genomes are 15,456 nucleotides in length, and phylogenetic analyses show them to be closely related.

## GENOME ANNOUNCEMENT

Avian paramyxoviruses (APMVs) are negative-stranded RNA viruses belonging to the genus *Avulavirus*, family *Paramyxoviridae*, and order *Mononegavirales* (1). There are 12 recognized serotypes (designated APMV-1 to APMV-12) (2). Two additional serotypes, APMV-13 and APMV-14, have been recently described (3, 4).

During a seabird health surveillance program in the Falkland Islands in 2007, oral and cloacal swabs and serum samples were collected from 193 rockhopper penguins (*Eudyptes chrysocome*). Four hemagglutinating agents were isolated from the tested samples. A hemagglutination inhibition assay determined that all four samples were negative when tested with antiserum against APMV-1, also known as Newcastle disease virus, but strongly positive against the homologous serum (5). The biological and serological characterization and partial sequences of all of these isolates suggested that they belong to a new serotype, APMV-10 (5). Here, we present the complete genome sequences of these four APMV-10 isolates, namely, APMV-10/penguin/Falkland Island/323/2007, APMV-10/penguin/Falkland Island/324/2007, APMV-10/penguin/Falkland Island/437/2007, and APMV-10/penguin/Falkland Island/539/2007.

Complete genome sequencing of the viruses was conducted at the Southeast Poultry Research Laboratory of the U.S. Department of Agriculture in Athens, GA. Viral RNA was isolated from allantoic fluid using the QIAamp viral RNA minikit (Qiagen, USA). The Illumina libraries for next-generation sequencing were prepared using the Kapa stranded RNA-Seq library preparation kit (Kapa Biosystems, Wilmington, MA), as per the manufacturer’s instructions. The distribution size and concentration of the prepared libraries were checked on a Bioanalyzer 2100, using the Agilent high-sensitivity (HS) DNA kit (Agilent Technologies, Germany), and a Qubit fluorometer, using the double-stranded DNA (dsDNA) HS assay kit (Life Technologies, Inc., USA), respectively. Paired-end sequencing (2 × 250 bp) was performed on an Illumina MiSeq instrument using the 500-cycle MiSeq reagent kit version 2 (Illumina, USA). Sequence data were assembled using MIRA version 3.4.1 (6) within a customized workflow on the Galaxy platform (7), as described previously (8). Missing positions at the 5′ and 3′ ends of the genomes were sequenced using Sanger technology, as described previously (9).

The complete genome sequences of each of the four APMV-10 isolates comprise 15,456 nucleotides (nt), complying with the paramyxovirus “rule of six” (10), and contain six open reading frames (3′-NP-P-M-F-HN-L-5′) of 1,374 nt, 1,218 nt, 1,100 nt, 1,611 nt, 1,728 nt, and 6,711 nt in length, respectively. The putative amino acid sequence of the fusion protein cleavage site of all four APMV-10 isolates was deduced as _96_KPSQRI_101_.

Phylogenetic analysis showed that compared to the other APMV serotypes, all four isolates grouped together, forming a unique branch, and they are most closely related to APMV-2 and APMV-8. The obtained data will facilitate further studies of avian paramyxoviruses.

### Accession number(s).

The complete genome sequences of the four APMV-10 isolates were deposited in GenBank under the accession numbers HM147142, HM755886, HM755887, and HM755888.
